# Unveiling Adatoms in On-Surface Reactions: Combining Scanning Probe Microscopy with van’t Hoff Plots

**DOI:** 10.1021/acs.jpcc.1c03134

**Published:** 2021-04-30

**Authors:** Juan Carlos Moreno-López, Alejandro Pérez Paz, Stefano Gottardi, Leonid Solianyk, Jun Li, Leticia Monjas, Anna K. H. Hirsch, Duncan John Mowbray, Meike Stöhr

**Affiliations:** † Faculty of Physics, University of Vienna, Boltzmanngasse 5, 1090 Vienna, Austria; ‡ Chemistry Department, United Arab Emirates University, 15551 Al Ain, United Arab Emirates; § Zernike Institute for Advanced Materials, University of Groningen, Nijenborgh 4, 9747 AG Groningen, The Netherlands; ∥ Stratingh Institute for Chemistry, University of Groningen, Nijenborgh 7, 9747 AG Groningen, The Netherlands; ⊥ School of Physical Sciences and Nanotechnology, Yachay Tech University, 100119 Urcuquí, Ecuador; # Helmholtz Institute for Pharmaceutical Research Saarland (HIPS)—Helmholtz Centre for Infection Research (HZI) and Department of Pharmacy, Saarland University, Campus Building E8.1, 66123 Saarbrücken, Germany

## Abstract

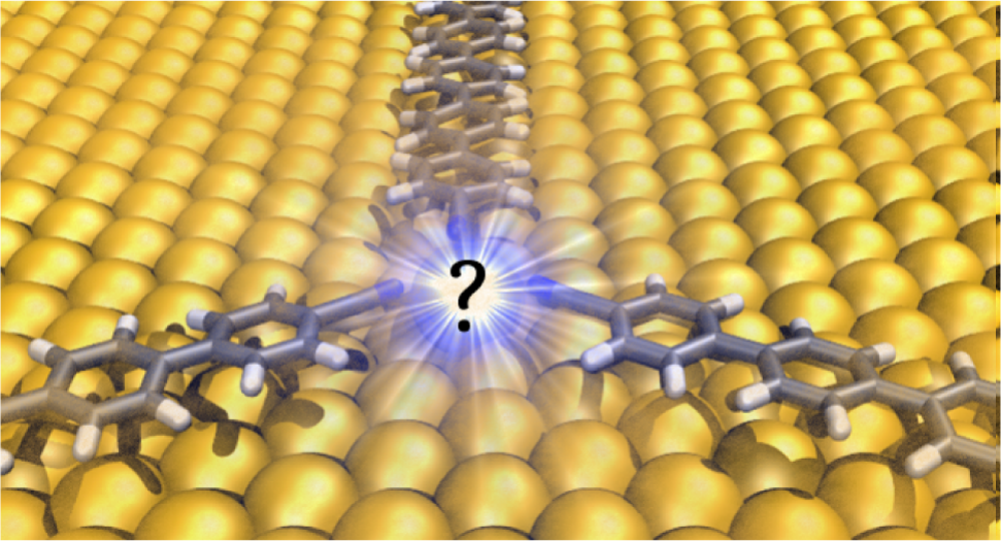

Scanning probe microscopy has become an essential tool to not only study pristine surfaces but also on-surface reactions and molecular self-assembly. Nonetheless, due to inherent limitations, some atoms or (parts of) molecules are either not imaged or cannot be unambiguously identified. Herein, we discuss the arrangement of two different nonplanar molecular assemblies of *para*-hexaphenyl-dicarbonitrile (Ph_6_(CN)_2_) on Au(111) based on a combined theoretical and experimental approach. For deposition of Ph_6_(CN)_2_ on Au(111) kept at room temperature, a rhombic nanoporous network stabilized by a combination of hydrogen bonding and antiparallel dipolar coupling is formed. Annealing at 575 K resulted in an irreversible thermal transformation into a hexagonal nanoporous network stabilized by native gold adatoms. However, the Au adatoms could neither be unequivocally identified by scanning tunneling microscopy nor by noncontact atomic force microscopy. By combining van’t Hoff plots derived from our scanning probe images with our density functional theory calculations, we were able to confirm the presence of the elusive Au adatoms in the hexagonal molecular network.

## 1 Introduction

Scanning probe microscopy has been developed over the past decades into an inherently valuable tool to gain insights into both surface-confined molecular self-assembly and on-surface reactions. For example, scanning tunneling microscopy (STM) has been routinely used to visualize organic molecules—even with submolecular resolution—for more than 30 years.^1−4^ As a result, profound knowledge has been obtained on molecular conformation, intermolecular bonding, and electronic properties of molecular self-assembly on surfaces. This knowledge enables the understanding and tuning of various processes, for example, on-surface reactions,^5−8^ molecular recognition,^9,10^ and molecular spin states,^11^ among others. However, since the tunneling current is mainly sensitive to the local density of states near the Fermi level, it might be quite challenging or even impossible to unequivocally identify all atoms in a molecule by STM. On the other hand, “qPlus”^12^ noncontact atomic force microscopy (nc-AFM), by probing the Pauli repulsion forces between the tip and sample, has been emerged as an enormously powerful tool to unveil the chemical structure of molecules in real-space based on the seminal work by Gross et al.^13^ This development has led to striking reports such as: bond-order discrimination,^14^ controlling the charge states of a single-molecular switch,^15^ chemical identification of individual surface atoms,^16^ and direct imaging of intramolecular changes originating from controlled on-surface reactions,^17,18^ just to mention a few. However, due to the nonmonotonic relationship between force and tip–sample distance, nc-AFM is usually recorded at constant height, parallel to the surface, making the imaging of nonplanar molecules or structures challenging. This is also true for surface-confined metal-organic coordination networks for which generally the adatoms are located closer to the surface than the molecules in the network.^19^ The main approach to overcome this limitation consists in acquiring multiple images at variable tip–sample distances.^20−22^ However, since the CO molecule at the tip apex exhibits a certain flexibility and thus can move, image artifacts have to be considered.^23−25^

Herein, we report an alternative way to circumventing the inherent limitations of STM and nc-AFM using a combination of van’t Hoff plots and density functional theory (DFT) calculations. The self-assembly of *para*-hexaphenyl-dicarbonitrile (Ph_6_(CN)_2_) on Au(111) investigated by means of “qPlus” nc-AFM, STM, and DFT serves as an instructive example for this. After deposition on Au(111) held at room temperature, Ph_6_(CN)_2_ mainly assembles into a rhombic network stabilized by a combination of hydrogen bonding and antiparallel dipolar coupling with the constituting molecules at two different vertical heights. Postdeposition annealing promotes the formation of a hexagonal Au-coordinated network. However, the coordinating Au adatoms could neither be identified with STM nor nc-AFM. With the help of van’t Hoff plots and DFT calculations, we are able to unambiguously confirm the presence of the elusive Au adatoms incorporated in the molecular network.

## 2 Methods

### 2.1 Experimental Details

The experiments were performed in an ultrahigh vacuum system with a base pressure of <1 × 10^–10^ mbar. Au(111) single crystals were prepared by repeated cycles of Ar^+^ sputtering and annealing at 700 K. *para*-Hexaphenyl-dicarbonitrile (Ph_6_(CN)_2_) molecules were synthesized according to previously reported procedures (see Figure S5 in the Supporting Information). Ph_6_(CN)_2_ was sublimated from a Knudsen cell at 570 K while the sample was kept at room temperature. A quartz crystal microbalance was used to monitor the deposition rate. The “qPlus” nc-AFM images were acquired at 4.5 K with a tuning fork sensor and a CO-functionalized tip. All bias voltages are with respect to the sample. The images were analyzed using the WSxM software.^26^

### 2.2 Computational Details of AFM Simulations

AFM simulations were performed with the “probe particle model” of Hapala et al.^23^ without electrostatic corrections. The simulation parameters were 0.5 N/m for the bending stiffness, an effective charge of 0.0 e was used for the probe particle, the amplitude was 0.5 Å, and we did scan steps of 0.05 Å.

### 2.3 GPAW/STM Calculations

DFT calculations were performed using linear combinations of atomic orbitals^27^ within the projector-augmented wave method (PAW)^28^ code GPAW.^29^ For the adsorbed species, we employed a double-zeta-polarized basis set and a single-zeta-polarized basis set for the Au(111) surface. We employed two different types of exchange and correlation (XC) functionals: the generalized gradient approximation for the XC functional as implemented by Perdew, Burke, and Ernzerhof (PBE),^30^ and vdW interactions at the Grimme’s D3 level (PBE-D3). STM simulations have employed the Tersoff–Hamann approximation^31^ in the constant-current mode with a bias of −1.5 V relative to the Fermi level as implemented in the code ASE.^32^

### 2.4 DFT Calculations of 1,4-Dicyanobenzene on Au(111)

We used the Quickstep (QS)^33^ module of the CP2K code.^34^ QS solves the electronic problem using a hybrid basis set approach that combines Gaussian and plane wave basis sets. The valence Kohn–Sham orbitals were expanded into a double-zeta-valence-polarized (DZVP) quality basis set (DZVP-MOLOPT-GTH for the adsorbate and MOLOPT-DZVP-SR-GTH for Au atoms), which is specifically optimized for its use with the GTH pseudopotentials.^35^ The valence electronic density was expanded using a plane-wave cutoff of 450 Ry. All CP2K calculations were carried out at the Γ point and employed an electronic Fermi–Dirac smearing temperature of 300 K (∼26 meV). Geometry relaxations were stopped once the maximum ionic force fell below 1.0 × 10^–3^ a.u. (0.0514 eV/Å). Only the adsorbate and the Au adatom were allowed to relax while the Au slab was frozen to the Cartesian coordinates derived from the experimental lattice constant of Au (*a* = 4.0782 Å). The PBE exchange correlation functional^30^ was supplemented with Grimme’s D3 van der Waals (vdW) corrections.^36^ Fifteen Å of vacuum and dipole were used to decouple the periodic images along the normal *z* direction.

## 3 Results and Discussion

**Figure 1:**
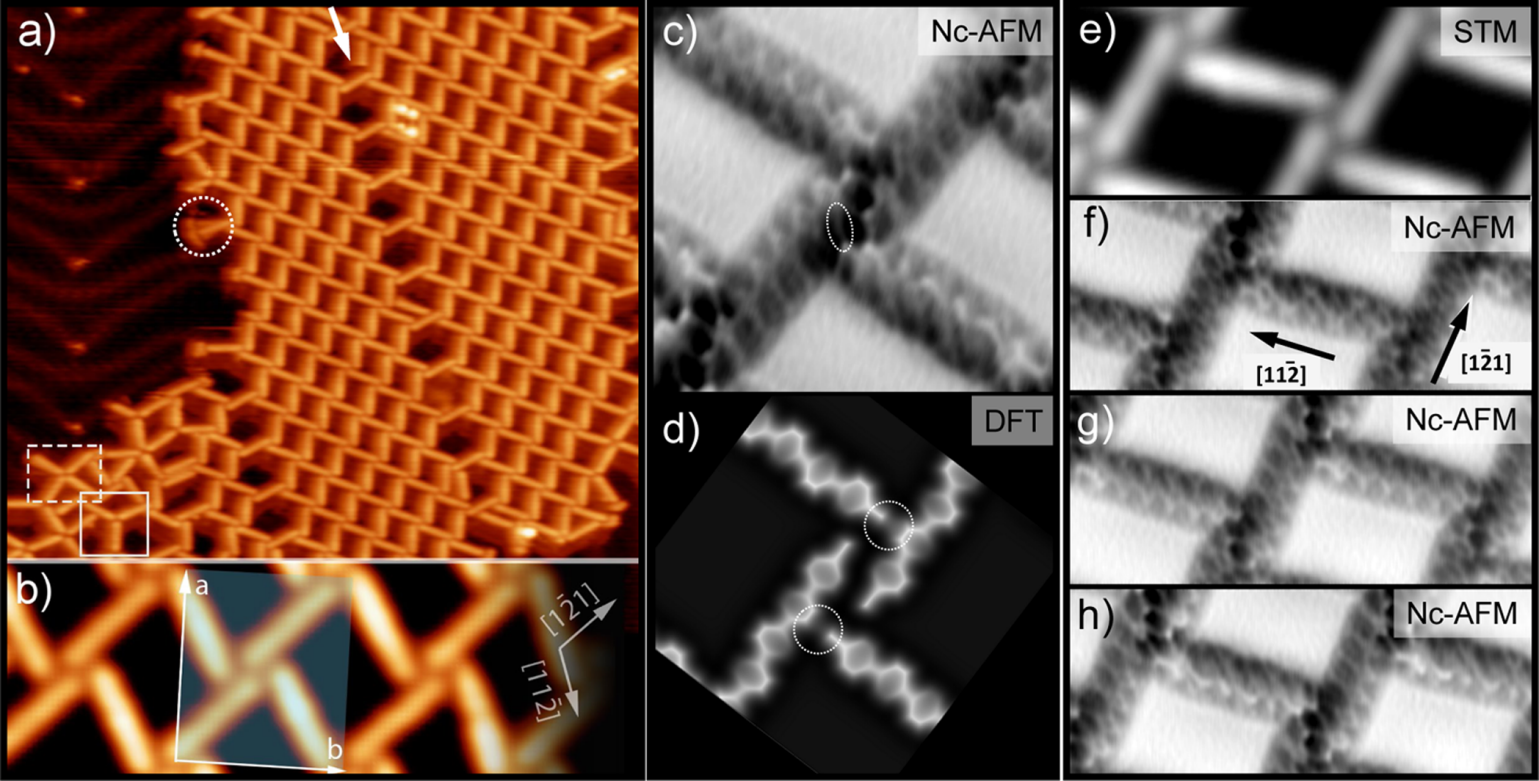
Rhombic network of Ph_6_(CN)_2_ on Au(111). (a) STM image. A trimer and tetramer are highlighted with continuous and dashed rectangles, respectively (*A* = 60 × 60 nm^2^, *U* = −1 V, *I* = 20 pA). (b) STM image. The semitransparent rectangle indicated the unit cell (*A* = 12 × 4 nm^2^, *U* = + 0.1 V, *I* = 150 pA). (c) Constant-height nc-AFM image (*A* = 4 × 4 nm^2^). (d) Simulated nc-AFM image. (e) STM image (*A* = 9.3 × 3.3 nm^2^, *U* = −0.9 V, *I* = 10 pA). (f–h) nc-AFM images acquired at decreasing tip–sample distances from f to h (*A* = 9.3 × 3.3 nm^2^).

After Ph_6_(CN)_2_ deposition on Au(111) held at room temperature, the samples were cooled down to 77 K to perform STM experiments. Single molecules can be easily identified by their rod-like shape, which is in good agreement with previous studies of Ph_6_(CN)_2_.^37,38^ For submonolayer coverages, ≈90% of the molecules were arranged into a rhombic network which contains four molecules per unit cell: two of them with their long axis running along the [112̅]_Au_ direction and the other two oriented along the [12̅1]_Au_ direction (see Figure 1a,b). The unit cell parameters of the rhombic network are determined to be *a* = 4.7 nm, *b* = 3.7 nm, and ϕ = 90°, in agreement with previous work on Ag(111).^37^ Remarkably, at this temperature, some molecules are still mobile at the boundaries of the network (see dotted circle in Figure 1a). The rhombic network is quasi-periodically perturbed either by a linear arrangement of quasi-hexagonal pores (see arrow in Figure 1a) or by the elbow sites of the Au(111) herringbone reconstruction (see Figure S1 in the Supporting Information). These observations suggest a rather weak physisorption of the molecules in the rhombic network.

A careful observation of Figure 1b,e unveils a brighter STM contrast for the molecules oriented along the [112̅]_Au_ direction. A similar behavior was reported for the rhombic network of Ph_6_(CN)_2_ on Ag(111), which could be attributed to different adsorption sites.^37^ However, since the STM current signal is based on a convolution of geometric and electronic contributions of the tip and sample, the interpretation has to be handled with care.^39^ To obtain deeper insights into the molecular conformation, nc-AFM measurements were performed. For this, the samples were cooled down to ≈4 K, and the tip apex was functionalized with a CO molecule, as previously reported.^13^ As shown in Figure 1c, Ph_6_(CN)_2_ molecules could be clearly imaged with submolecular resolution. The six phenyl rings as well as the terminal cyano groups at both ends of the molecules can now be easily identified. A closer inspection of Figure 1c unveils the presence of several bonding-like features between neighboring molecules (see Figure S2 in the Supporting Information). Nonetheless, these features should not be directly interpreted as real intermolecular bonds, since the origin of this contrast is still under debate.^23,24,40,41^

To obtain additional information, we performed AFM simulations using the “probe particle model”.^23^Figure 1d shows our simulated AFM image in good agreement with our experimental ones, both in the dimensions and the overall appearance. However, contrary to the experimental data, the AFM simulation does not show the bonding-like features, suggesting that these features are imaging artifacts, which occur due to the lateral relaxation of the CO molecule. Further support for this hypothesis is given by the fact that the lengths of some of the bonding-like features notably exceed the expected values for C≡N···H–C hydrogen bonds, that is, 2–3 Å (see Figure S2 in the Supporting Information).^42−45^ In particular, the bonding-like feature marked by the white oval in Figure 1c is measured to be 4.1 Å, which is considerably too long to be identified as a hydrogen bond. In contrast to the sharp bonding-like features observed in the experiments, AFM simulations display a more extended region of increased electron density, highlighted by dotted circles in Figure 1d. These larger interaction regions are in good agreement with the interaction between a proton acceptor group and an organic ring system, previously reported as the proton-acceptor ring interaction.^44^ In addition, the antiparallel orientation of the cyano groups leads to further interactions between the molecules oriented along the [112̅]_Au_ direction. From electrostatics, the interaction energy between two local point dipoles in vacuum is given by
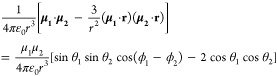
1where **r** is the vector connecting the centers of the electric dipoles, θ_*i*_ is the polar angle between dipoles **μ_*i*_**, and ϕ_*i*_ is the azimuthal angle between the dipoles, with *i* = 1, 2. For the case of two identical dipoles (μ_1_ = μ_2_ = μ) in an antiparallel coplanar orientation (ϕ_1_ – ϕ_2_ = ±180°), the previous expression reduces to . Using the experimental value for the dipole moment of benzonitrile (μ ∼ 4.18 D)^46^ and a typical distance *r* ∼ 5.9 Å between the nitrile terminations of opposing precursors in the rhombic network, we estimate that the side-by-side (θ_1_ = θ_2_ = 90°) antiparallel dipole arrangement is stabilized by , which is twice the thermal energy at 300 K (0.6 kcal/mol) and significantly more than the one available in our STM measurements at 77 K. Therefore, this stabilization is large enough to “lock” the antiparallel arrangement of dipoles at lower temperatures as seen for other adsorbed nitriles^45,47−49^ and other dipolar compounds.^50,51^

To further study the molecular conformation in the rhombic network, we have performed nc-AFM measurements at decreasing tip–sample distances (see Figure 1f–h). While in Figure 1f, the molecules oriented in the [12̅1]_Au_ direction are barely discernible surrounded by a strong dark halo, the molecules oriented in the [112̅]_Au_ direction were clearly resolved. In contrast, upon reducing the tip–sample distance (see Figure 1h), the molecules oriented in the [12̅1]_Au_ direction are clearly discernible, whereas the molecules oriented in the [112̅]_Au_ direction appear distorted. Figure 1g was obtained for an intermediate tip–sample distance, between the one of Figure 1f,h, for which all the molecules are discernible. It should be noted that the high-resolved nc-AFM contrast arises from repulsive Pauli forces between the functionalized tip and the molecules, whereas the dark featureless halo is mostly due to attractive vdW forces.^13,52^ Therefore, when the molecule–tip distance is large, the Pauli contribution to the nc-AFM image becomes negligible resulting in the imaging of a featureless dark halo. On the contrary, when the molecule–tip distance is too small, the dominant repulsive Pauli forces induce a lateral displacement of the CO molecule attached to the tip apex, resulting in a distorted image. Based on these considerations, we infer that the molecules oriented along the [12̅1]_Au_ direction are closer to the surface than the molecules oriented in the [112̅]_Au_ direction, explaining—at least partially—the contrast difference observed in our STM measurements.

**Figure 2:**
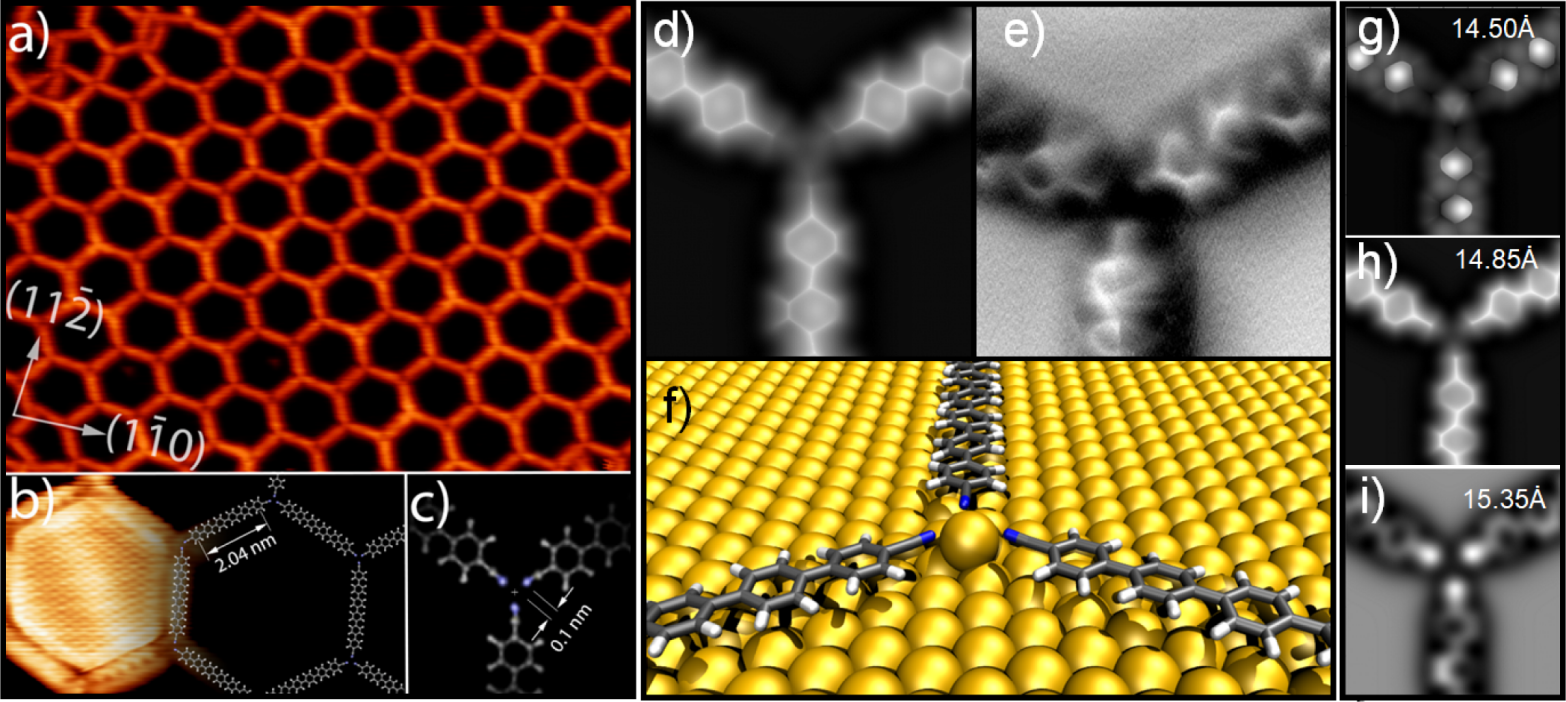
(a) STM images of Ph_6_(CN)_2_ on Au(111) after postdeposition thermal annealing at 575 K (*A* = 45 × 35 nm^2^; *U* = 2 V; *I* = 20 pA). (b) High-resolution STM image (*A* = 7.5 × 6 nm^2^; *U* = 0.01 V; *I* = 40 pA). (c) Schematic representation of the molecular binding motif. (d) Simulated nc-AFM image of the hexagonal network. (e) Nc-AFM image of the hexagonal network acquired with a CO-terminated tip (*A* = 2.5 × 2.5 nm^2^). (f) Relaxed structure from DFT calculations. (g–i) Simulated nc-AFM images of the hexagonal network at (g) 14.50, (h) 14.85, and (i) 15.35 Å from the bottom layer of the Au(111) surface slab.

In addition, even for samples without postdeposition annealing, some molecules were observed to have their cyano groups pointing against each other (see the trimer and tetramer highlighted with rectangles in Figure 1a). The occurrence of these bonding motifs increased when postdeposition annealing was done, resulting in a net decrease in the surface covered by the rhombic network, that is, the number of trimers increased while the number of molecules forming the rhombic network decreased (see Figure S3 in the Supporting Information). After annealing the samples at 575 K, Ph_6_(CN)_2_ molecules self-assembled into a hexagonal network where the cyano groups of three molecules are pointing directly toward each other (see Figure 2a). This kind of interaction is energetically unfavorable due to the electrostatic repulsion between N atoms facing each other. Therefore, it is usually assumed that these bonding motifs are stabilized by coordinating metallic atoms.^38^ Moreover, by performing the same electrostatic considerations as for the rhombic network, the hexagonal network would be destabilized by about twice the thermal energy at 300 K and would require CN–Au bonds for stabilization. However, if we assume the presence of a gold adatom involved in metal–ligand bonding to the cyano groups of three Ph_6_(CN)_2_ molecules, the distance between the terminal nitrogen atoms and the expected coordinating gold atom would be only 0.10 nm, about half the distance expected for metal–ligand interactions (see Figure 2c).^38,45,53,54^ Moreover, our high-resolution STM images do not show distinctive signs which could be unambiguously attributed to coordinating Au adatoms at the center of the junctions (see Figure 2b). It is worth highlighting that the herringbone reconstruction was not observed to be either lifted or perturbed, neither for the case of individual trimers nor for the hexagonal network. At this point, the experiments seem to provide contradictory arguments regarding the presence of coordinating Au adatoms in the hexagonal network. On the one hand, the Au adatoms are required from a rational point of view to avoid electrostatic repulsion between N atoms by stabilizing the hexagonal network via metal–ligand interactions. On the other hand, the distances obtained from the STM data suggest that the bond distance between the gold adatom and the cyano groups is too small.

To gain additional insights into the plausible presence of Au adatoms, we performed nc-AFM measurements and DFT calculations. Figure 2d shows our nc-AFM simulation of the trimer motif stabilized by a Au adatom where the overall appearance of Ph_6_(CN)_2_ molecules, including the alternate twisting of the phenyl rings, is in excellent agreement with experiments (Figure 2e). Remarkably, Figure 2d does not show any feature indicative of a Au adatom at the center of the junction, which can be clearly observed in the relaxed DFT structure shown in Figure 2f, which was used to perform our nc-AFM simulations. Figure 2f shows the subtle bending of the cyano groups sideways and upward from the surface, explaining the short distance observed between the terminal nitrogen atoms and the Au adatom when a planar arrangement of molecules and adatoms is assumed. In fact, our DFT calculations find that the Au adatom is 0.8 Å below the molecular plane, which is more than sufficient to inhibit detection by nc-AFM.

As previously reported and observed for our rhombic network, the Pauli contribution to nc-AFM images is highly sensitive to the tip–sample distance.^13,19^Figure 2g–i shows AFM simulations for our DFT-relaxed structures of the hexagonal network for increasing tip–sample distance, that is, the tip–sample distances increase from Figure 2g–i. As shown by the simulations, when it is possible to observe a faint contrast due to the Au adatom, the Ph_6_(CN)_2_ molecules are imaged with inverted contrast due to the strong tip–molecule interaction at this shorter distance (Figure 2g). As the tip–sample distance increases, the molecules start to be clearly resolved but bright contrast from the Au adatom fades away to the point that it is not visible anymore (see Figure 2g–i and Video S1 with 0.05 Å steps per frame in the Supporting Information). Therefore, it is not possible to simultaneously obtain clear images of the adsorbates and the Au adatoms, which is a common problem in standard nc-AFM measurements.

**Figure 3:**
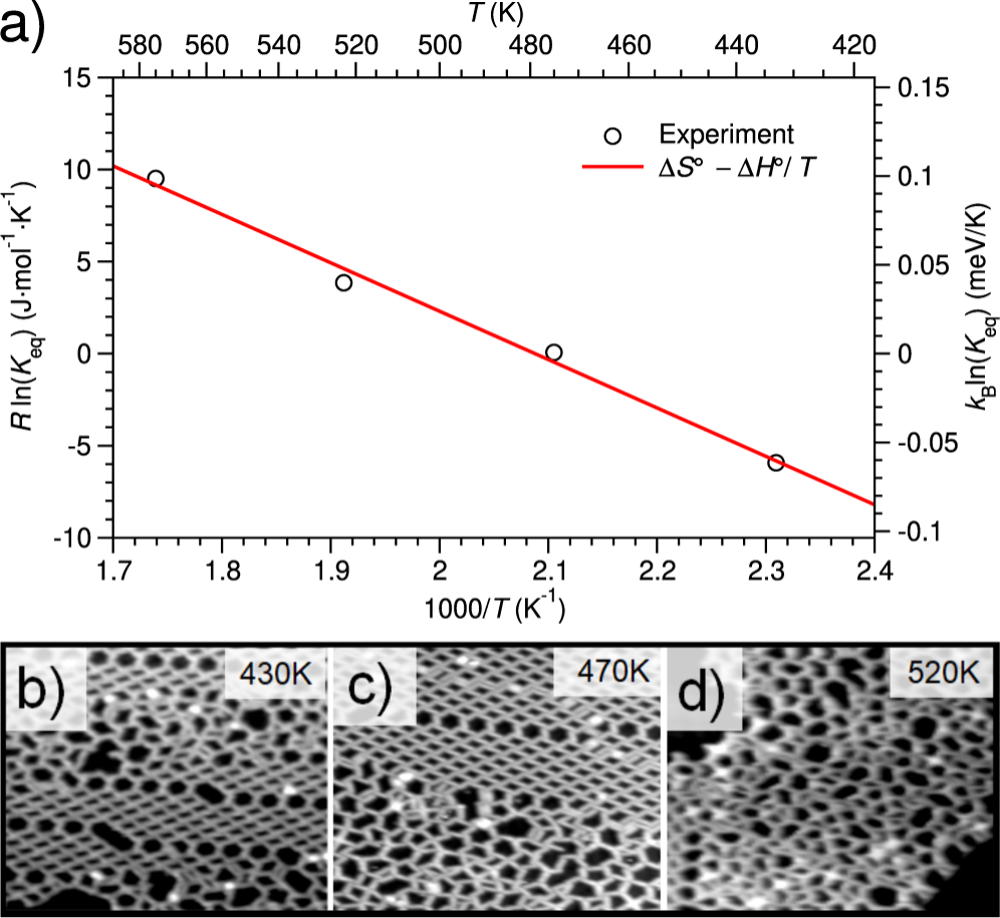
(a) Van’t Hoff plot of the rhombic to trimer metalation reaction, eq 2, comparing our experimental measurements (circles) to the linear fit Δ*S*° – Δ*H*°/*T* (red line *r* = 99.6%) of eq 4 yields a standard reaction entropy of Δ*S*° ≈ 55 J/(mol·K) ≈0.57 meV/K and enthalpy of Δ*H*° ≈ 26.3 kJ/mol ≈0.272 eV; (b–d) prototypical STM images showing the increase in disorder as a function of the postdeposition annealing temperature.

It is clear by now that the coordinating Au adatoms cannot be unambiguously identified by nc-AFM or STM. In order to circumvent this limitation, we propose that the rhombic network based on fourfold junctions stabilized mainly by dipole–dipole interaction between antiparallel −CN groups is thermally transformed into the hexagonal network featuring threefold junctions based on metal–ligand interactions according to the following reaction:
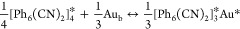
2where [Ph_6_(CN)_2_]_4_ and [Ph_6_(CN)_2_]_3_ are the basic bonding motifs for the junctions in the rhombic and hexagonal networks, respectively, “*” denotes adsorption on the Au(111) surface, and Au_b_ is a gold atom in the bulk. Next, we performed a statistical analysis (over 1400 bonding motifs were analyzed) (see Figures 3b–d and S3 in the Supporting Information). By counting the numbers of rhombic and hexagonal junctions as a function of the temperature, we were able to estimate the equilibrium constant *K*_eq_ of the reaction given by eq 2
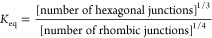
3

The plot of ln(*K*_eq_) versus 1/*T*, better known as the van’t Hoff plot, is given in Figure 3a and follows a linear behavior (Pearson correlation coefficient *r* = 99.6%) corresponding to the van’t Hoff equation
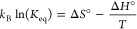
4where Δ*S*° and Δ*H*° are the standard reaction entropy and enthalpy, in eV/K and eV, respectively, which are assumed to be temperature-independent. From the slope of the linear fit (−37930.8 ± 2395.9 K), we determine a metalation standard enthalpy Δ*H*° = 3.27 eV, which amounts to 0.27 eV/molecule in the threefold junction, in excellent agreement with our DFT estimate using the PBE functional (0.28 eV/molecule).^30^ The positive value of Δ*H*° indicates that under standard conditions, the metalation reaction (eq 2) is endothermic and is dominated by the energy cost of forming Au adatoms on the surface. The formation of Au adatoms on Au(111) was reported to be endothermic with a formation energy of 0.67 eV,^55^ which agrees well with our own PBE value of 0.78 eV.

**Figure 4:**
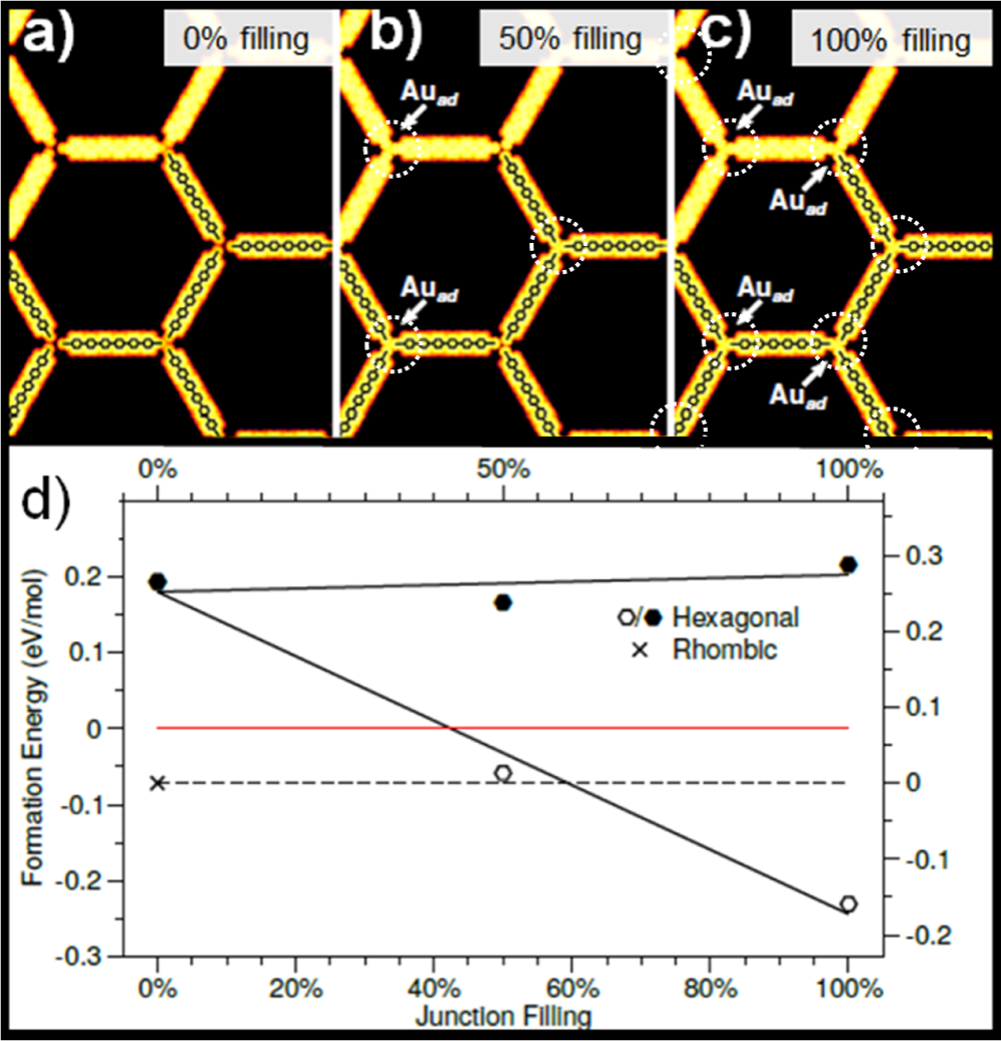
a–c) STM simulations of the hexagonal network with 0, 50, and 100% of junctions filled with Au adatoms. (d) Formation energies in eV/mol relative to isolated Au adatoms and molecules for rhombic network (X), hexagonal network (empty hexagons), and Au adatoms (solid hexagons). The energy scale relative to the rhombic network is added at the right side of the plot. The adatoms are marked with dotted circles in the figure (a–c).

In order to further confirm the presence of the Au adatom within the hexagonal network, we calculated the DFT energetic and simulated STM images with and without gold adatoms. Figure 4a–c shows STM simulations with no Au adatoms present at the junctions (0% filling), half present (50% filling), and fully present (100% filling). A careful observation of Figure 4a–c shows Au adatoms appearing as tiny spherical protrusions while their absence results in a small but discernible gap at the “empty” junctions. Figure 4d shows the formation energy as a function of the Au adatom filling of the hexagonal network, including the energy cost of Au adatom formation from the bulk. As shown in Figure 4d, a hexagonal network without Au adatoms (0% filling) is less stable than the rhombic network by ≈0.27 eV/molecule. On the contrary, when Au adatoms are present in all the junctions (100% filling), the hexagonal network is more stable than the rhombic network by ≈0.17 eV/molecule. An equilibrium in energy between a partially filled hexagonal network and the rhombic network is found for ≈60% filling. The irreversible transformation of the rhombic network toward the hexagonal network observed in our experiments allows us to confirm the presence of Au adatoms in the hexagonal network to form a more stable arrangement than the rhombic one. However, at finite temperatures, the minimum of free energy is reached after introducing some degree of disorder/defects into the hexagonal overlayer where the presence of some empty junctions cannot be ruled out.

As a proof-of-concept and inspired by previous works,^45^ we study the thermodynamic stability of a metalated trimer formed by a simpler dinitrile precursor molecule, 1,4-dinitrilebenzene (Ph(CN)_2_), on Au(111). Our DFT calculations show that this organometallic compound lies flat at 3.39 Å above the Au(111) surface, with 3 equivalent N–Au bonds of 2.395 Å at almost perfect 120° from each other. The Au adatom sits at 1.16 Å below the molecular trimer plane which explains why it is so difficult to identify the presence of Au adatoms in experimental nc-AFM (see Figure S4 in the Supporting Information). The formation energy for the metalated trimer is −0.95 eV and it is therefore energetically stable. Removing the central Au adatom causes that the monomers repel each other and the whole trimer decomposes. Remarkably, without the Au adatom, the monomers spontaneously reorient their dipoles and rearrange themselves into a “frustrated” structure which resembles the rhombic structure of Ph_6_(CN)_2_ (see Video S2 in the Supporting Information). Last but not the least, it is worth mentioning that DFT calculations on the Ph(CN)_2_ use a different computational protocol (code, basis sets, etc.) than DFT on Ph_6_(CN)_2_, which adds robustness to our conclusions regarding the metalation of the hexagonal network.

## 4 Conclusions

We have studied the rhombic to hexagonal thermal transformation of Ph_6_(CN)_2_ deposited on Au(111). We found that both STM and nc-AFM are not enough to unequivocally identify the presence of Au adatoms at the hexagonal junctions. Using van’t Hoff plots and DFT calculations, we were able to prove the presence of Au adatoms that stabilize the hexagonal network. Remarkably, from the van’t Hoff plots, we obtained a metalation standard enthalpy of 0.27 eV/molecule at the hexagonal junction in excellent agreement with our DFT estimation (0.28 eV/molecule). This work shows that by a combined analysis of scanning probe microscopy and van’t Hoff plots, it is possible to confirm the presence of the elusive Au adatoms stabilizing the hexagonal network. We believe that this method can likely be extended to a wide range of on-surface reactions.

## Supporting Information Available

Details of DFT functional, rhombic network versus square network comparison, additional STM images, entropy considerations, additional scanning probe images, and synthesis of Ph_6_(CN)_2_ (PDF)
Nc-AFM simulated images at variable tip–sample distance recorded with a 0.05 Å tip–sample distance difference per frame (AVI)
DFT calculation of a metalated trimer of Ph(CN)_2_ after removing the central Au adatom (MPG)

## Supplementary Material

jp1c03134_si_001.pdf

jp1c03134_si_002.avi

jp1c03134_si_003.mpg

## References

[ref1] Ohtani H.; Wilson R. J.; Chiang S.; Mate C. M. Scanning tunneling microscopy observations of benzene molecules on the Rh (111)-(3× 3)(C 6 H 6+ 2CO) surface. Phys. Rev. Lett. 1988, 60, 2398 10.1103/physrevlett.60.2398. 10038341

[ref2] Spong J. K.; Mizes H. A.; LaComb L. J. Jr.; Dovek M. M.; Frommer J. E.; Foster J. S. Contrast mechanism for resolving organic molecules with tunnelling microscopy. Nature 1989, 338, 137–139. 10.1038/338137a0.

[ref3] Foster J. S.; Frommer J. E. Imaging of liquid crystals using a tunnelling microscope. Nature 1988, 333, 542–545. 10.1038/333542a0.

[ref4] Gimzewski J. K.; Stoll E.; Schlittler R. R. Scanning tunneling microscopy of individual molecules of copper phthalocyanine adsorbed on polycrystalline silver surfaces. Surf. Sci. 1987, 181, 267–277. 10.1016/0039-6028(87)90167-1.

[ref5] Liu X.-H.; Guan C.-Z.; Ding S.-Y.; Wang W.; Yan H.-J.; Wang D.; Wan L.-J. On-surface synthesis of single-layered two-dimensional covalent organic frameworks via solid–vapor interface reactions. J. Am. Chem. Soc. 2013, 135, 10470–10474. 10.1021/ja403464h. 23786481

[ref6] Eichhorn J.; Heckl W. M.; Lackinger M. On-surface polymerization of 1, 4-diethynylbenzene on Cu (111). Chem. Commun. 2013, 49, 2900–2902. 10.1039/c3cc40444g. 23459835

[ref7] Moreno-López J. C.; Grizzi O.; Sánchez E. A. Thermal Stability of N, N’-Bis (1-ethylpropyl) perylene-3, 4, 9, 10-tetracarboxdiimide Films on Cu (100). J. Phys. Chem. C 2016, 120, 19630–19635. 10.1021/acs.jpcc.6b04157.

[ref8] Moreno-López J. C.; Fedi F.; Argentero G.; Carini M.; Chimborazo J.; Meyer J.; Pichler T.; Mateo-Alonso A.; Ayala P. Exclusive Substitutional Nitrogen Doping on Graphene Decoupled from an Insulating Substrate. J. Phys. Chem. C 2020, 124, 22150–22157. 10.1021/acs.jpcc.0c06415. PMC7552092 33072238

[ref9] Otero R.; Hümmelink F.; Sato F.; Legoas S. B.; Thostrup P.; Lægsgaard E.; Stensgaard I.; Galvão D. S.; Besenbacher F. Lock-and-key effect in the surface diffusion of large organic molecules probed by STM. Nat. Mater. 2004, 3, 779–782. 10.1038/nmat1243. 15502831

[ref10] Bonifazi D.; Mohnani S.; Llanes-Pallas A. Supramolecular chemistry at interfaces: molecular recognition on nanopatterned porous surfaces. Chem.—Eur. J. 2009, 15, 7004–7025. 10.1002/chem.200900900. 19569139

[ref11] Miyamachi T.; Gruber M.; Davesne V.; Bowen M.; Boukari S.; Joly L.; Scheurer F.; Rogez G.; Yamada T. K.; Ohresser P.; et al. Robust spin crossover and memristance across a single molecule. Nat. Commun. 2012, 3, 938 10.1038/ncomms1940. 22760637

[ref12] Giessibl F. J. High-speed force sensor for force microscopy and profilometry utilizing a quartz tuning fork. Appl. Phys. Lett. 1998, 73, 3956–3958. 10.1063/1.122948.

[ref13] Gross L.; Mohn F.; Moll N.; Liljeroth P.; Meyer G. The chemical structure of a molecule resolved by atomic force microscopy. Science 2009, 325, 1110–1114. 10.1126/science.1176210. 19713523

[ref14] Gross L.; Mohn F.; Moll N.; Schuler B.; Criado A.; Guitián E.; Peña D.; Gourdon A.; Meyer G. Bond-order discrimination by atomic force microscopy. Science 2012, 337, 1326–1329. 10.1126/science.1225621. 22984067

[ref15] Leoni T.; Guillermet O.; Walch H.; Langlais V.; Scheuermann A.; Bonvoisin J.; Gauthier S. Controlling the charge state of a single redox molecular switch. Phys. Rev. Lett. 2011, 106, 216103 10.1103/physrevlett.106.216103. 21699320

[ref16] Sugimoto Y.; Pou P.; Abe M.; Jelinek P.; Pérez R.; Morita S.; Custance Ó. Chemical identification of individual surface atoms by atomic force microscopy. Nature 2007, 446, 64–67. 10.1038/nature05530. 17330040

[ref17] de Oteyza D. G.; Gorman P.; Chen Y.-C.; Wickenburg S.; Riss A.; Mowbray D. J.; Etkin G.; Pedramrazi Z.; Tsai H.-Z.; Rubio A.; et al. Direct imaging of covalent bond structure in single-molecule chemical reactions. Science 2013, 340, 1434–1437. 10.1126/science.1238187. 23722428

[ref18] Riss A.; Wickenburg S.; Gorman P.; Tan L. Z.; Tsai H.-Z.; de Oteyza D. G.; Chen Y.-C.; Bradley A. J.; Ugeda M. M.; Etkin G.; et al. Local electronic and chemical structure of oligo-acetylene derivatives formed through radical cyclizations at a surface. Nano Lett. 2014, 14, 2251–2255. 10.1021/nl403791q. 24387223 PMC4022646

[ref19] Riss A.; Richter M.; Paz A. P.; Wang X.-Y.; Raju R.; He Y.; Ducke J.; Corral E.; Wuttke M.; Seufert K.; et al. Polycyclic aromatic chains on metals and insulating layers by repetitive [3+ 2] cycloadditions. Nat. Commun. 2020, 11, 1490 10.1038/s41467-020-17857-3. 32198456 PMC7083871

[ref20] Moreno C.; Stetsovych O.; Shimizu T. K.; Custance O. Imaging three-dimensional surface objects with submolecular resolution by atomic force microscopy. Nano Lett. 2015, 15, 2257–2262. 10.1021/nl504182w. 25756297

[ref21] Schuler B.; Zhang Y.; Collazos S.; Fatayer S.; Meyer G.; Pérez D.; Guitián E.; Harper M. R.; Kushnerick J. D.; Peña D.; et al. Characterizing aliphatic moieties in hydrocarbons with atomic force microscopy. Chem. Sci. 2017, 8, 2315–2320. 10.1039/c6sc04698c. 28451335 PMC5363392

[ref22] Telychko M.; Su J.; Gallardo A.; Gu Y.; Mendieta-Moreno J. I.; Qi D.; Tadich A.; Song S.; Lyu P.; Qiu Z.; et al. Strain-Induced Isomerization in One-Dimensional Metal–Organic Chains. Angew. Chem. 2019, 131, 18764–18770. 10.1002/ange.201909074. 31608578

[ref23] Hapala P.; Kichin G.; Wagner C.; Tautz F. S.; Temirov R.; Jelínek P. Mechanism of high-resolution STM/AFM imaging with functionalized tips. Phys. Rev. B: Condens. Matter Mater. Phys. 2014, 90, 085421 10.1103/physrevb.90.085421.

[ref24] Hämäläinen S. K.; van der Heijden N.; van der Lit J.; den Hartog S.; Liljeroth P.; Swart I. Intermolecular contrast in atomic force microscopy images without intermolecular bonds. Phys. Rev. Lett. 2014, 113, 186102 10.1103/physrevlett.113.186102. 25396382

[ref25] Labidi H.; Koleini M.; Huff T.; Salomons M.; Cloutier M.; Pitters J.; Wolkow R. A. Indications of chemical bond contrast in AFM images of a hydrogen-terminated silicon surface. Nat. Commun. 2017, 8, 14222 10.1038/ncomms14222. 28194036 PMC5316802

[ref26] Horcas I.; Fernández R.; Gómez-Rodríguez J. M.; Colchero J.; Gómez-Herrero J.; Baro A. M. WSXM: a software for scanning probe microscopy and a tool for nanotechnology. Rev. Sci. Instrum. 2007, 78, 013705 10.1063/1.2432410. 17503926

[ref27] Larsen A. H.; Vanin M.; Mortensen J. J.; Thygesen K. S.; Jacobsen K. W. Localized Atomic Basis Set in the Projector Augmented Wave Method. Phys. Rev. B: Condens. Matter Mater. Phys. 2009, 80, 195112 10.1103/physrevb.80.195112.

[ref28] Mortensen J. J.; Hansen L. B.; Jacobsen K. W. Real-Space Grid Implementation of the Projector Augmented Wave Method. Phys. Rev. B: Condens. Matter Mater. Phys. 2005, 71, 035109 10.1103/physrevb.71.035109.

[ref29] Enkovaara J.; Rostgaard C.; Mortensen J. J.; Chen J.; Dułak M.; Ferrighi L.; Gavnholt J.; Glinsvad C.; Haikola V.; Hansen H. A.; et al. Electronic Structure Calculations with GPAW: A Real-Space Implementation of the Projector Augmented-Wave Method. J. Phys.: Condens. Matter 2010, 22, 253202 10.1088/0953-8984/22/25/253202. 21393795

[ref30] Perdew J. P.; Burke K.; Ernzerhof M. Generalized Gradient Approximation Made Simple. Phys. Rev. Lett. 1996, 77, 3865 10.1103/physrevlett.77.3865. 10062328

[ref31] Tersoff J.; Hamann D. R. Theory of the scanning tunneling microscope. Phys. Rev. B: Condens. Matter Mater. Phys. 1985, 31, 805–813. 10.1103/physrevb.31.805. 9935822

[ref32] Larsen A. H.; Mortensen J. J.; Blomqvist J.; Castelli I. E.; Christensen R.; Duł M.; Friis J.; Groves M. N.; Hammer B.; Hargus C.; et al. The atomic simulation environment—a Python library for working with atoms. J. Phys.: Condens. Matter 2017, 29, 273002 10.1088/1361-648x/aa680e. 28323250

[ref33] VandeVondele J.; Krack M.; Mohamed F.; Parrinello M.; Chassaing T.; Hutter J. Quickstep: Fast and accurate density functional calculations using a mixed Gaussian and plane waves approach. Comput. Phys. Commun. 2005, 167, 103–128. 10.1016/j.cpc.2004.12.014.

[ref34] Hutter J.; Iannuzzi M.; Schiffmann F.; VandeVondele J. cp2k: atomistic simulations of condensed matter systems. Wiley Interdiscip. Rev.: Comput. Mol. Sci. 2014, 4, 15–25. 10.1002/wcms.1159.

[ref35] Goedecker S.; Teter M.; Hutter J. Separable dual-space Gaussian pseudopotentials. Phys. Rev. B: Condens. Matter Mater. Phys. 1996, 54, 1703 10.1103/physrevb.54.1703. 9986014

[ref36] Grimme S.; Antony J.; Ehrlich S.; Krieg H. A consistent and accurate ab initio parametrization of density functional dispersion correction (DFT-D) for the 94 elements H-Pu. J. Chem. Phys. 2010, 132, 154104 10.1063/1.3382344. 20423165

[ref37] Kühne D.; Klappenberger F.; Decker R.; Schlickum U.; Brune H.; Klyatskaya S.; Ruben M.; Barth J. V. Self-assembly of nanoporous chiral networks with varying symmetry from sexiphenyl-dicarbonitrile on Ag (111). J. Phys. Chem. C 2009, 113, 17851–17859. 10.1021/jp9041217.

[ref38] Kühne D.; Klappenberger F.; Decker R.; Schlickum U.; Brune H.; Klyatskaya S.; Ruben M.; Barth J. V. High-Quality 2D Metal- Organic Coordination Network Providing Giant Cavities within Mesoscale Domains. J. Am. Chem. Soc. 2009, 131, 3881–3883. 10.1021/ja809946z. 19256496

[ref39] Tersoff J.; Hamann D. R. Theory and application for the scanning tunneling microscope. Phys. Rev. Lett. 1983, 50, 1998 10.1103/physrevlett.50.1998.

[ref40] Pavliček N.; Herranz-Lancho C.; Fleury B.; Neu M.; Niedenführ J.; Ruben M.; Repp J. High-resolution scanning tunneling and atomic force microscopy of stereochemically resolved dibenzo [a, h] thianthrene molecules. Phys. Status Solidi B 2013, 250, 2424–2430. 10.1002/pssb.201349229.

[ref41] Guo C.-S.; Xin X.; Van Hove M. A.; Ren X.; Zhao Y. Origin of the contrast interpreted as intermolecular and intramolecular bonds in atomic force microscopy images. J. Phys. Chem. C 2015, 119, 14195–14200. 10.1021/acs.jpcc.5b02649.

[ref42] Zhang J.; Chen P.; Yuan B.; Ji W.; Cheng Z.; Qiu X. Real-space identification of intermolecular bonding with atomic force microscopy. Science 2013, 342, 611–614. 10.1126/science.1242603. 24072819

[ref43] Steiner T. Donor and acceptor strengths in C–H··· O hydrogen bonds quantified from crystallographic data of small solvent molecules. New J. Chem. 1998, 22, 1099–1103. 10.1039/a804121k.

[ref44] Arras E.; Seitsonen A. P.; Klappenberger F.; Barth J. V. Nature of the attractive interaction between proton acceptors and organic ring systems. Phys. Chem. Chem. Phys. 2012, 14, 15995–16001. 10.1039/c2cp42293j. 23089650

[ref45] Okuno Y.; Yokoyama T.; Yokoyama S.; Kamikado T.; Mashiko S. Theoretical study of benzonitrile clusters in the gas phase and their adsorption onto a Au (111) surface. J. Am. Chem. Soc. 2002, 124, 7218–7225. 10.1021/ja011744v. 12059248

[ref46] Haynes W. M. CRC Handbook of Chemistry and Physics, 95th ed.; CRC Press LLC: Boca Raton: FL, 2014; pp 9–53.

[ref47] Müller K.; Moreno-López J. C.; Gottardi S.; Meinhardt U.; Yildirim H.; Kara A.; Kivala M.; Stöhr M. Cyano-Functionalized Triarylamines on Coinage Metal Surfaces: Interplay of Intermolecular and Molecule–Substrate Interactions. Chem.—Eur. J. 2016, 22, 581–589. 10.1002/chem.201503205. 26636437

[ref48] Gottardi S.; Müller K.; Moreno-López J. C.; Yildirim H.; Meinhardt U.; Kivala M.; Kara A.; Stöhr M. Cyano-Functionalized Triarylamines on Au (111): Competing Intermolecular versus Molecule/Substrate Interactions. Adv. Mater. Interfaces 2014, 1, 1300025 10.1002/admi.201300025.

[ref49] Ceccatto dos Santos A.; de Campos Ferreira R. C.; Moreno-López J. C.; Barreto L.; Lepper M.; Landers R.; Steinrück H.-P.; Marbach H.; de Siervo A. Cyano-Functionalized Porphyrins on Cu (111) from One-Dimensional Wires to Two-Dimensional Molecular Frameworks: On the Role of Co-Deposited Metal Atoms. Chem. Mater. 2020, 32, 2114–2122. 10.1021/acs.chemmater.9b05256.

[ref50] de Oteyza D. G.; Pérez Paz A.; Chen Y.-C.; Pedramrazi Z.; Riss A.; Wickenburg S.; Tsai H.-Z.; Fischer F. R.; Crommie M. F.; Rubio A. Noncovalent dimerization after enediyne cyclization on Au (111). J. Am. Chem. Soc. 2016, 138, 10963–10967. 10.1021/jacs.6b05203. 27490459

[ref51] Moreno-López J. C.; Mowbray D. J.; Pérez Paz A.; de Campos Ferreira R. C.; Ceccatto dos Santos A.; Ayala P.; de Siervo A. Roles of Precursor Conformation and Adatoms in Ullmann Coupling: An Inverted Porphyrin on Cu (111). Chem. Mater. 2019, 31, 3009–3017. 10.1021/acs.chemmater.9b00668.

[ref52] Moll N.; Gross L.; Mohn F.; Curioni A.; Meyer G. The mechanisms underlying the enhanced resolution of atomic force microscopy with functionalized tips. New J. Phys. 2010, 12, 125020 10.1088/1367-2630/12/12/125020.

[ref53] Faraggi M. N.; Jiang N.; Gonzalez-Lakunza N.; Langner A.; Stepanow S.; Kern K.; Arnau A. Bonding and charge transfer in metal–organic coordination networks on Au (111) with strong acceptor molecules. J. Phys. Chem. C 2012, 116, 24558–24565. 10.1021/jp306780n.

[ref54] Przychodzen P.; Korzeniak T.; Podgajny R.; Sieklucka B. Supramolecular coordination networks based on octacyanometalates: from structure to function. Coord. Chem. Rev. 2006, 250, 2234–2260. 10.1016/j.ccr.2006.01.026.

[ref55] Wang J.-g.; Selloni A. The c (4× 2) structure of short-and intermediate-chain length alkanethiolate monolayers on Au (111): a DFT study. J. Phys. Chem. C 2007, 111, 12149–12151. 10.1021/jp0745891.

