# Supplementary material for: Unveiling Adatoms in On-Surface Reactions: Combining Scanning Probe Microscopy with van’t Hoff Plots
Source: J Phys Chem C Nanomater Interfaces. 2021 Apr 30;125(18):9847–54. doi: 10.1021/acs.jpcc.1c03134 (PMC8279638; doi:10.1021/acs.jpcc.1c03134)
Supplement: Supplementary file 1 — jp1c03134_si_001.pdf [file jp1c03134_si_001.pdf]

Supplementary Information for: Unveiling Adatoms in On-Surface Reactions: Combining Scanning Probe Microscopy with Van't Hoff Plots

Juan Carlos Moreno-López,[*,†] Alejandro Pérez Paz,[‡] Stefano Gottardi,[¶] Leonid Solianyk,[¶] Jun Li,[¶] Leticia Monjas,[§] Anna K. H. Hirsch,[§,⊥] Duncan John Mowbray,[‖] and Meike Stöhr[*,¶]

†*Faculty of Physics, University of Vienna, Boltzmanngasse 5, 1090 Vienna, Austria.*

‡*Chemistry Department, United Arab Emirates University, 15551, Al Ain, United Arab Emirates.*

¶*Zernike Institute for Advanced Materials, University of Groningen, Nijenborgh 4, 9747 AG Groningen, The Netherlands.*

§*Stratingh Institute for Chemistry. University of Groningen, Nijenborgh 7, 9747 AG Groningen, The Netherlands.*

‖*School of Physical Sciences and Nanotechnology. Yachay Tech University, 100119 Urcuquí, Ecuador.*

⊥*Helmholtz Institute for Pharmaceutical Research Saarland (HIPS) - Helmholtz Centre for Infection Research (HZI) and Department of Pharmacy, Saarland University, Campus building E8.1, 66123, Saarbrücken, Germany*

E-mail: juan.moreno@univie.ac.at; m.a.stohr@rug.nl

This supplementary information provides additional data and explanations to complement the understanding of the work entitled Elusive gold adatoms identified by van´t Hoff plots and DFT calculations: Dicarbonitrile-hexaphenyl on Au(111)

DFT functional

When vdW interactions are neglected (PBE)[1] the rhombic network has only a weak binding energy of 0.071 eV/molecule, in qualitative agreement with the experiments where the rhombic network can not be imaged at room temperature by STM and even at 77 K some molecules are still mobile at its boundaries (See Fig. 1a in the main manuscript). However, if vdW interactions are included at the Grimme's D3 level (PBE-D3),[2] the rhombic network becomes considerably more stable, ≈ 0.2 eV/molecule, which corresponds to 0.8 eV per junction but does not agree with our experimental observation, being PBE the best functional to describe the energetics of $Ph_6(CN)_2$ on Au(111). We attribute this artifact to the overestimation of the Grimme's vdW attractions which artificially lowers the energy of the assembly with higher number of intermolecular contacts (rhombic).

Rombic network vs square network

Herein, we studied the possibility that $Ph_6(CN)_2$ molecules self-assembly into a Square network instead of the experimentally observed rhombic network. We have found that a rhombic network of dipoles ($\theta_1 = 0°, \theta_2 \sim 60°$) is preferred over a rectangular network ($\theta_1 = 0°, \theta_2 = 90°$) because the former is lower in energy whereas the latter is energetically neutral (See equation 1 in main manuscript). In this qualitative discussion we have ignored other effects such as van der Waals interactions and image dipole effects from the metal substrate.

Entropy considerations

From the intersect of the linear fit with the X axis in Fig. 3 in the main manuscript (79.2 ± 4.9) we determine a standard reaction entropy of 6.82 meV/K, which amounts to an entropy gain of 0.57 meV/K per precursor at the 3-fold junction. This minimal entropy increase could be attributed to the *"soft"* nature of the Au–NC interaction. Another contribution to the entropy gain would be the likely presence of defects in the hexagonal overlayer, as shown in Fig. 3 in the main manuscript. We further speculate that not all the 3-fold junctions could be metalated, which would add an

entropy increase according to Boltzmann equation $\Delta S = k_B \ln 2$. This would be relevant at lower T's where the availability of Au adatoms make them *limiting reagants*.

References

1. Perdew, J. P.; Burke, K.; Ernzerhof, M. Generalized gradient approximation made simple. *Phys. Rev. Lett.* **1996**, *77*, 3865.

2. Grimme, S.; Antony, J.; Ehrlich, S.; Krieg, H. A consistent and accurate ab initio parametrization of density functional dispersion correction (DFT-D) for the 94 elements H-Pu. *J. Chem. Phys.* **2010**, *132*, 154104.

3. Farahat, A. A.; Kumar, A.; Say, M.; Barghash, A. E.-D. M.; Goda, F. E.; Eisa, H. M.; Wenzler, T.; Brun, R.; Liu, Y.; Mickelson, L., *et al.* Synthesis, DNA binding, fluorescence measurements and antiparasitic activity of DAPI related diamidines. *Bioorganic & medicinal chemistry* **2010**, *18*, 557–566.

4. Yu, S.; Saenz, J.; Srirangam, J. K. Facile synthesis of N-aryl pyrroles via Cu (II)-mediated cross coupling of electron deficient pyrroles and arylboronic acids. *J. Org. Chem.* **2002**, *67*, 1699–1702.

5. Kühne, D.; Klappenberger, F.; Decker, R.; Schlickum, U.; Brune, H.; Klyatskaya, S.; Ruben, M.; Barth, J. V. High-Quality 2D Metal- Organic Coordination Network Providing Giant Cavities within Mesoscale Domains. *J. Am. Chem. Soc.* **2009**, *131*, 3881–3883.

Additional Figures and videos

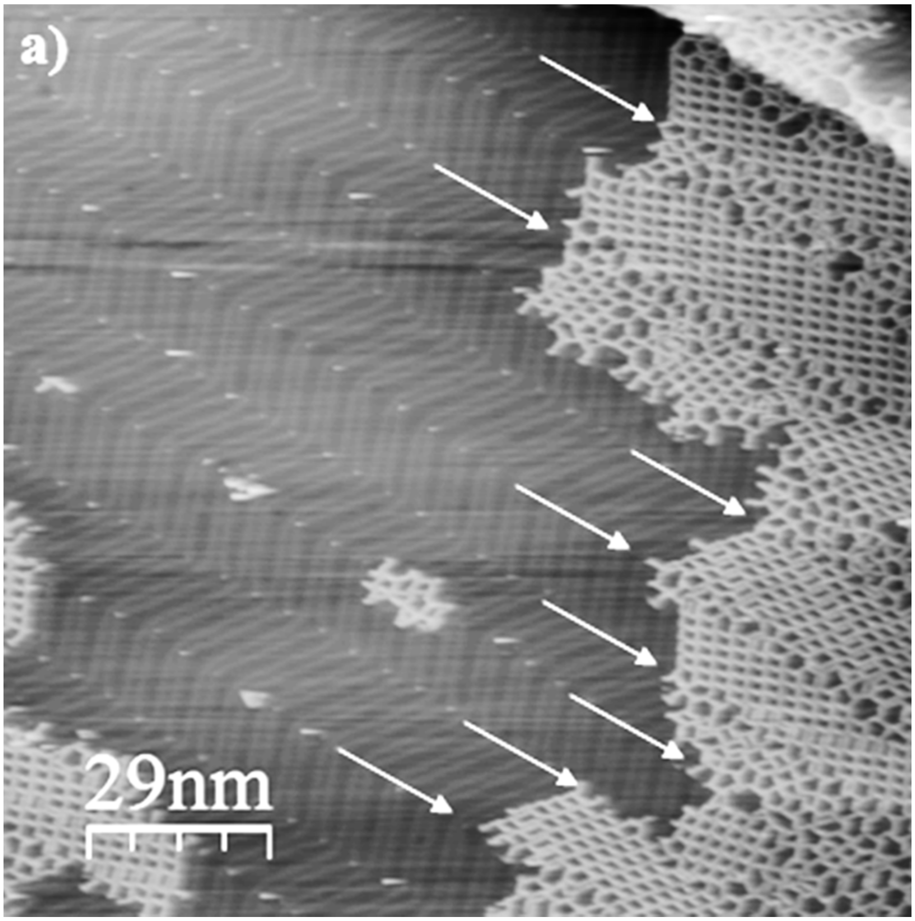

Figure S 1: STM image of the rhombic network. The white arrows show places where the rhombic network is perturbed for the elbows sites of the herringbone reconstruction (A = 150×150 nm^2; U = -1.8 V; I = 20 pA; T = 77 K)

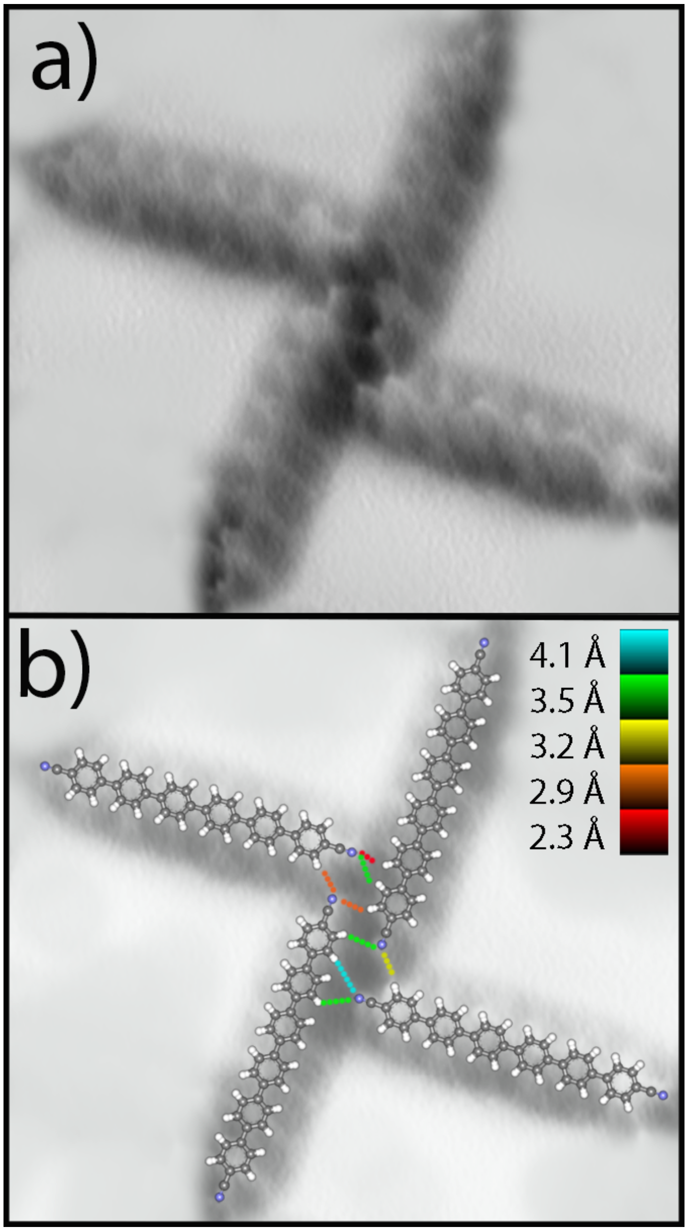

Figure S 2: a) Constant-height nc-AFM image of the four-fold bonding motif of the rhombic network acquired with a CO terminated tip. (4 × 4 nm2; VB = 0.0 V; TSTM = 5 K. b) Superposition of the 4-fold bonding motif with the molecular structures. The red dashed oval indicates a bonding-like feature that greatly exceeds the typical lenght for hydrogen bonding.(A = 4×4 nm^2; U = 0.0 V; T = 5 K) .

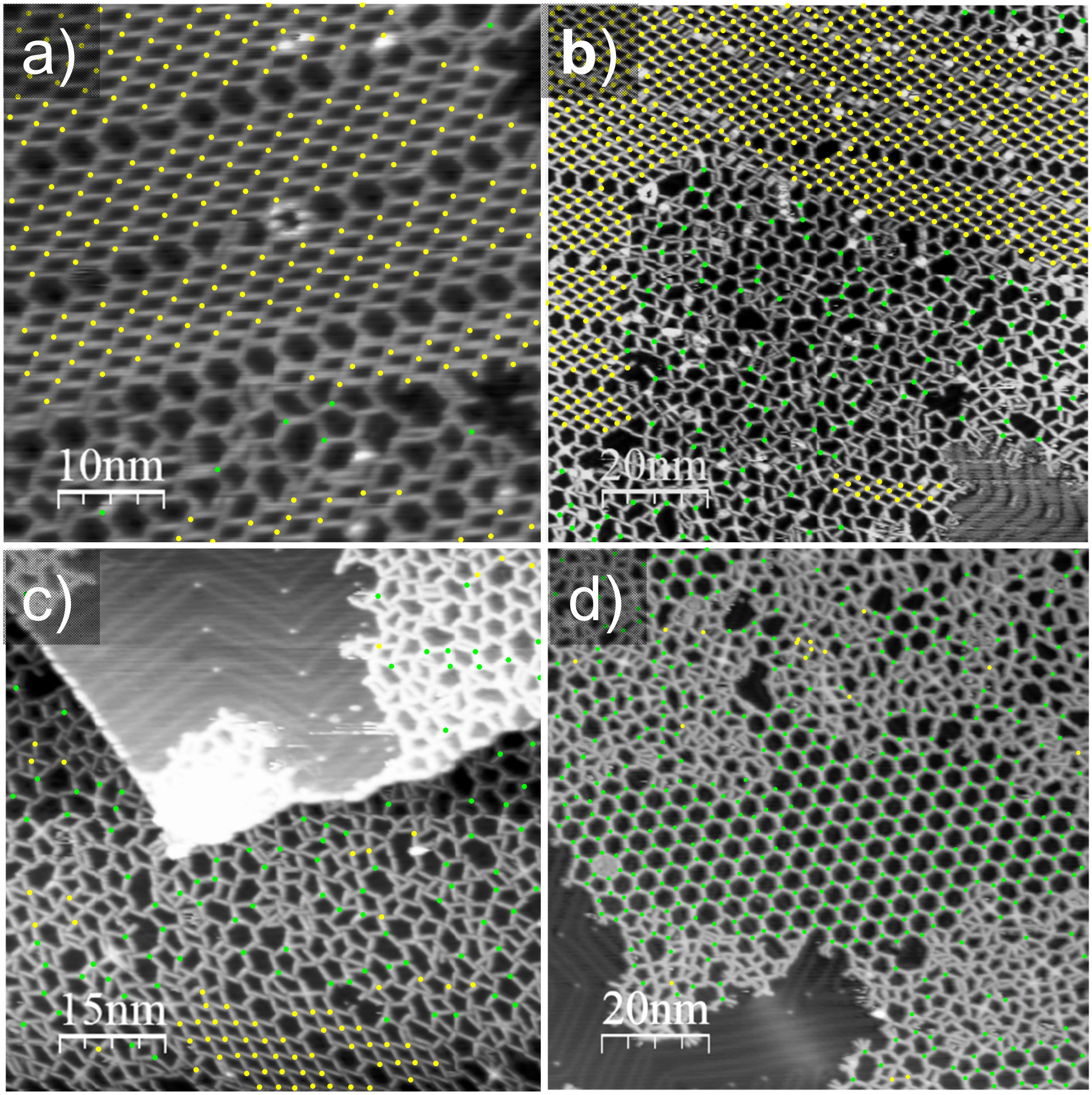

Figure S 3: STM images after performing post-deposition annealing treatment at: a)433 K, b) 475 K., c) 523 K and d)575 K. The yellow and green circles indicate the rhombic and hexagonal junctions, respectively

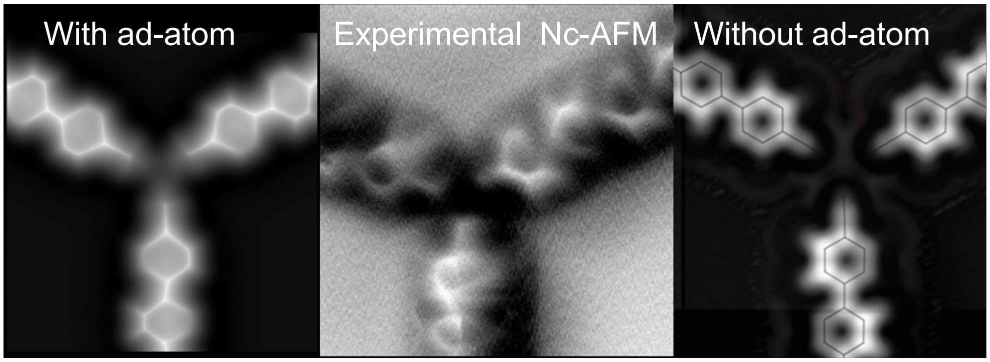

Figure S 4: Nc-AFM images of the hexagonal network. left: DFT simulated image whith Au adatom; center: Experimental image; left: DFT simulated image without Au adatom.

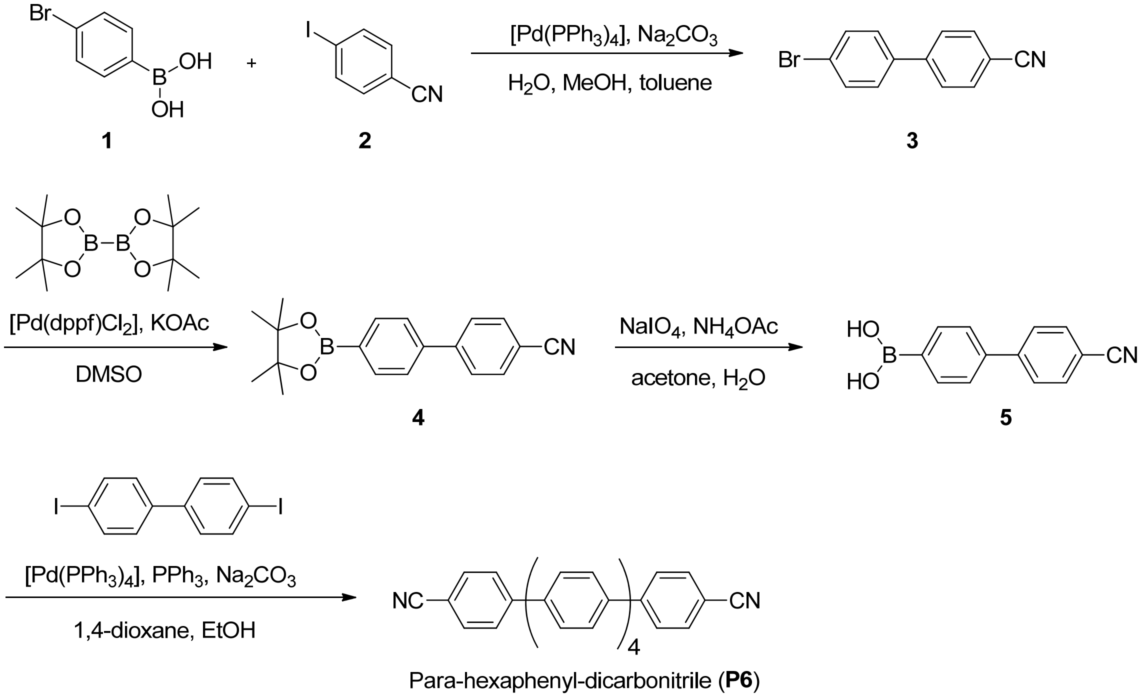

Figure S 5: Synthesis of *para*-hexaphenyl-dicarbonitrile (P6): 4-Bromo-4’-cyanobiphenyl (3),[3] 4’-(4,4,5,5-tetramethyl-1,3,2-dioxaborolan-2-yl)-biphenyl-4-carbonitrile (4),[4] 4’-cyano-4-biphenylboronic acid (5)[4] and [1,4’;1’,1”;4”,1’”;4’”,1””;4””,1””’]sexiphenyl-4-4””’-dicarbonitrile (P6)[5] were synthesized according to previously reported procedures.
